# Metabolic syndrome, depression, and fibromyalgia syndrome prevalence in patients with irritable bowel syndrome: A case-control study

**DOI:** 10.1097/MD.0000000000020577

**Published:** 2020-06-05

**Authors:** Muharrem Bayrak

**Affiliations:** Department of Internal Medicine, University of Health Sciences Erzurum Regional Training and Research Hospital Erzurum, Turkey.

**Keywords:** anxiety, depression, insulin resistance, irritable bowel syndrome, metabolic syndrome, obesity

## Abstract

Although both metabolic syndrome (MetS) and irritable bowel syndrome (IBS) have been linked with altered gut microbiota, only a few studies investigated the association between them. Hence, we aimed to evaluate the prevalence of MetS along with depression and fibromyalgia syndrome (FMS) in IBS patients.

This was a case-control study in which 3808 consecutive patients who attended outpatient clinics of Erzurum Regional Training and Research Hospital between May 2019 and August 2019 were evaluated in terms of IBS with Rome-IV criteria. Out of 486 patients who were diagnosed as IBS, 176 patients were excluded for various reasons. Control subjects were randomly selected from IBS-negative subjects. MetS was diagnosed based on International Diabetes Federation criteria. Depression, anxiety disorder, and FMS were assessed via Hamilton Depression Scale, Beck Anxiety Inventory, and American College of Rheumatology criteria, respectively. Blood samples were obtained to measure biochemical parameters.

Study group included 310 IBS patients, and control group included 304 subjects. The prevalence of the MetS was significantly higher among IBS patients compared with controls (36.8% vs 21.7%, respectively, *P* = .006). The rate of obesity was 18.1% among IBS subjects, and 10.2% in the controls. The prevalence of fibromyalgia (30% vs 3%, respectively, *P* < .001), anxiety-disorder (39.7% vs 10.2%, *P* < .001) and depression (8.1% vs 4.9%, *P* < .001) were significantly higher in IBS group than controls.

Metabolic syndrome and obesity were significantly more frequent in IBS patients compared with controls. FMS, anxiety disorder, and depression were also more common among IBS patients.

## 1 Introduction

Irritable bowel syndrome (IBS) is the most commonly encountered gastrointestinal disorder.^[1]^ It is a diagnosis of exclusion and is characterized by the presence of recurrent abdominal pain or discomfort along with altered bowel habits. Despite its high prevalence, exact pathogenesis is yet to be fully elucidated. Several mechanisms have been suggested, among which are dysmotility, increased visceral sensation, impaired brain-gut interaction, and psychosocial distress. However, with the increasing popularity of gut microbiota, some authors also speculated that IBS might be linked to abnormalities in gut microbiota. Some studies reported that the frequency of small bacterial overgrowth is increased in IBS patients and, vice versa.^[2,3]^ Moreover, some intervention studies, in which antibiotics or probiotics were used, showed significant improvements in IBS symptoms.^[4,5]^

Metabolic syndrome (MetS) is defined as the presence of ≥3 of the following criteria: increased waist circumference, elevated triglycerides, low high-density lipoprotein (HDL) cholesterol, hypertension, and elevated fasting blood glucose level.^[6]^ Notably, metabolic syndrome has actually become a global epidemic, with as high as 40% prevalence in some regions.^[7]^ Obesity and insulin resistance are considered to play the most significant role in the pathogenesis of the metabolic syndrome.^[8]^ Interestingly, accumulating clinical and experimental evidence has shown an association between the gut microbiome and some components of metabolic syndrome.^[9]^

It is surprising that despite their considerably high prevalence in the general population, and their increasingly reported association with gut microbiota, only a few studies to date have attempted to elucidate the potential link between IBS and MetS.^[10,11]^ We hypothesized that since gut microbiota plays a role to some extent in IBS and some components of metabolic syndrome, IBS, through changes in gut microbiome, might be a risk factor for the development of the metabolic syndrome. Hence, we aimed to evaluate the association of IBS and MetS in adult patients in a case-control study design. We also sought to evaluate the prevalence of fibromyalgia syndrome (FMS) and depression, other frequent disorders accompanying IBS, in this study.

## 2 Patients and methods

### 2.1 Study subjects and design

**Figure 1:**
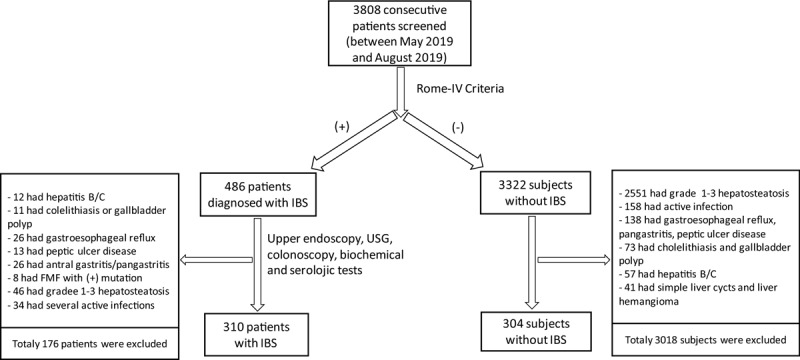
Flow-chart showing study design.

This was a cross-sectional case-control study in which 3808 consecutive patients who attended outpatient clinics of Erzurum Regional Training and Research Hospital between May 2019 and August 2019 were evaluated for eligibility for inclusion in the study. Turkish versions of the Bristol Stool Chart and Rome-IV criteria were completed by face-to-face interviews and recorded for each patient. Overall, 486 patients (12.7%) were diagnosed with IBS. All patients who deemed to have IBS underwent esophagogastroduodenoscopy, colonoscopy, and abdominal ultrasonography as well as biochemical and serologic tests, including anti-HBs antigen and anti-HCV antibody. Patients with the following diagnoses were excluded from the study: positive HBs antigen, positive anti-HCV antibody, gastrointestinal and other malignancies, intestinal obstruction and other intestinal disorders, cholelithiasis and gall bladder polyps, gastric and duodenal ulcers, gastroesophageal reflux, esophagitis, cirrhosis, and other liver diseases, systemic infection and inflammatory disorders. One hundred and seventy-six patients were excluded due to several reasons. After application of exclusion criteria, 310 patients were included in the study. Study patients were divided into four IBS subtypes based on the features of the Bristol Stool Chart as constipation-predominant IBS, diarrhea-predominant IBS, mixed IBS, and unsubtyped IBS groups. Remaining 3322 patients in whom IBS was not found were evaluated with the same diagnostic tests, and a total of 3018 subjects were excluded due to various reasons. In the final analysis, 304 control subjects were included in the study. Study flow-chart is depicted in Figure 1.

The Clinical Research Ethics Board of Health Sciences University, Erzurum Regional Training and Research Hospital approved the study protocol (2019/08–68). All study participants completed written informed consent forms before enrollment. All study procedures were performed in compliance with the Declaration of Helsinki.

### 2.2 Data collection and Anthropometric measurements

Detailed medical history, including education status, cigarette smoking, marital status, presence of hypertension and/or diabetes mellitus, and statin use was obtained for each study participant. A psychiatrist blinded to the status of patient grouping performed the psychiatric assessment for all patients and control subjects. All study participants underwent a physical examination for fibromyalgia assessment by the same physician. Systolic and diastolic blood pressure was measured while sitting on a chair with arm-support after a 30-minute rest by an automatic sphygmomanometer (Braun BUA7200). Blood pressure was calculated as the mean of 2 measurements performed 15 minutes apart. Venous blood samples were drawn for measurement of fasting plasma glucose, hemoglobin A1c, alanine aminotransferase, aspartate aminotransferase, triglycerides, low-density lipoprotein (LDL) cholesterol, HDL cholesterol, total cholesterol, albumin, thyroid stimulating hormone, ferritin, and vitamin B12. The weight and height of all study participants were measured on an empty stomach and with light clothing. Body mass index (BMI) was calculated as the weight divided by height squared (kg/m^2^). Waist circumference was measured at the level of the umbilicus. Homeostasis model assessment for insulin ratio (HOMA-IR) was calculated using serum glucose and insulin values [HOMA-IR = Fasting plasma glucose (mg/dL) X Fasting plasma insulin level (μU/mL)].

### 2.3 Definitions of IBS, metabolic syndrome, depression, anxiety disorder, and fibromyalgia syndrome

International Diabetes Federation criteria were used for the definition of metabolic syndrome.^[12]^ Patients were classified as having MetS if 2 or more of the following 4 criteria were fulfilled in addition to abdominal obesity (waist circumference ≥94 cm in males and >80 cm in females):

1. Elevated serum triglycerides level (≥150 mg/dL),
2. low HDL cholesterol level (<50 mg/dL in males, and <40 mg/dL in females),
3. hypertension (blood pressure ≥130/85 mmHg) and
4. presence of type 2 diabetes mellitus or fasting blood glucose level ≥100 mg/dL.

We used ethnically adjusted (Europids) waist circumference cutoff values as ≥94 cm in men and ≥80 cm in women. Obesity and overweight were defined as BMI values ≥30 and 25 to 29.9 kg/m^2^, respectively.

Beck Anxiety Inventory was used to evaluate the presence of anxiety disorder. Beck Anxiety Inventory is a 21-question self-administered questionnaire that is used for measuring the degree of anxiety in adults.^[13]^ Patients scored 8 or higher were accepted as having anxiety disorder. Hamilton Depression Rating Scale (HDRS) was used to evaluate depressive symptoms of the whole study cohort. HDRS is one of the most extensively used depression assessment scales. It is administered by the physician.^[14]^ Patients who had ≥7 total points were accepted as having a depressive disorder.

Fibromyalgia syndrome was diagnosed as per the American College of Rheumatology 2013 criteria. Basic laboratory tests to exclude other disorders as the source of pain were performed, including vitamin B12 level, thyroid function tests, and vitamin D level.

### 2.4 Statistical analysis

To summarize study data, descriptive statistics were reported in continuous variables as either mean ± standard deviation or median-interquartile range depending on the distribution type of the data. Categorical variables were reported as numbers and respective percentages. The normality check of numerical variables was performed via the Kolmogorov-Smirnov test. According to groups, to compare categorical variables, chi-square/Fisher exact test used, while Independent Samples *t* test or Mann–Whitney *U* tests were used for continuous variables in case of normal and nonnormal distribution, respectively. In the comparison of some laboratory and clinical parameters according to IBS subgroups, One Way ANOVA and Kruskal–Wallis tests were used when numerical variables normally and nonnormally distributed, respectively. Jamovi (2018, Version 1.0.7, retrieved from https://www.jamovi.org) and JASP Team (2018, Version 0.10.2) were used to perform the statistical analyses. A *P*-value of <.05 was considered for statistical significance. The *P*-value is the probability of obtaining the respective test results assuming that the null hypothesis is correct (that there is no significant difference between the groups). For comparisons based on IBS type, Bonferroni correction was applied in order not to increase type-I error (*P* = .05 / 4 = 0.0125). Univariate and multiple logistic regression models were used to investigate possible risk factors affecting the development of metabolic syndrome, and the results were given as 95% confidence interval with odds ratio.

### 2.5 Power analysis

Since there was not any closely similar study in the literature, we performed a preliminary study with 10 cases in each group to be able to determine the magnitude of the effect size (the amount of difference between the groups). Then, only descriptive statistics that directed at the status of the development of "metabolic syndrome” were calculated to estimate the magnitude of the effect size. According to the power analysis based on the descriptive statistics derived from this study, to be able to detect the statistical significance of a 20% difference in terms of the frequency of "metabolic syndrome” between the groups with 80% power and 5% type I error, at least 106 subjects (a total of 206 subjects) were aimed to be included in each group (Table 1).

**Table 1:**
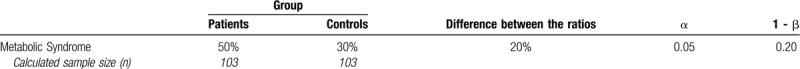
Results of power analysis.

On the other hand, we calculated the power of the study on the data derived from the study (310 patients and 304 control subjects) as 95% (Post Hoc Power Analysis). Power analysis was performed by means of G∗Power 3.1.9.4 for Windows (*Open Source*) package program.

## 3 Results

### 3.1 Baseline characteristics and laboratory data

Overall, 614 subjects participated in the study. While the study group comprised 310 IBS patients, the control group included 304 age and gender-matched subjects. The prevalence of IBS among the whole group of the screened patients was 12.7%. The mean age of the study and control groups was 37.9 ± 12.1 and 37.7 ± 12.2 years, respectively. The groups were comparable in terms of mean age and gender distribution. Table 2 summarizes the baseline characteristics of the IBS and the control groups. The IBS group had significantly higher education status compared with the control group. Liver transaminase levels were significantly higher among IBS subjects than control subjects, though the values were still in the normal laboratory reference range.

**Table 2:**
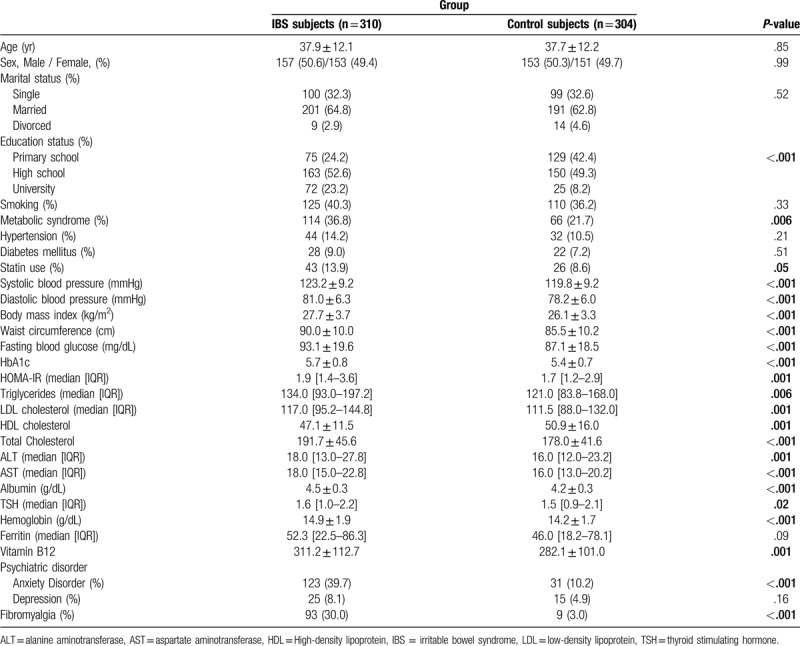
Baseline clinicodemographic and laboratory characteristics of the IBS and control groups.

Among IBS subjects, the most common subtype was the constipation-predominant (n = 142, 23.1%). Diarrhea-predominant IBS, mixed IBS, and unsubtyped IBS rates were 9%, 12.2%, and 6.2%, respectively (Table 3). The prevalence of metabolic syndrome was not different among IBS subgroups. None of the studied laboratory parameters, anthropometric measurements, or demographic and clinical characteristics was different across IBS subgroups.

**Table 3:**
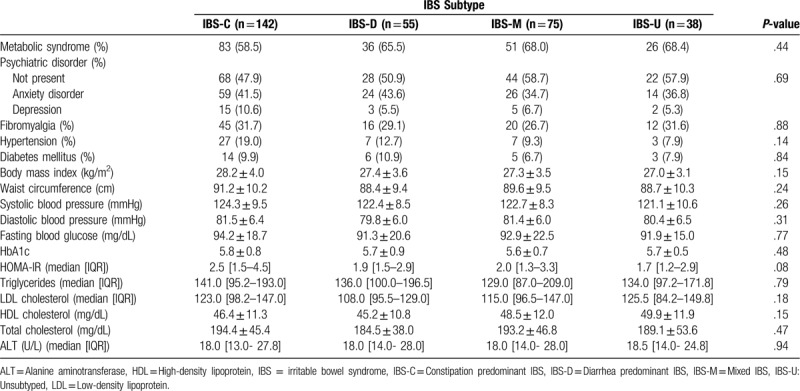
Metabolic syndrome frequency and selected laboratory data in IBS subtypes.

### 3.2 Metabolic syndrome (MetS) and its components

**Figure 2:**
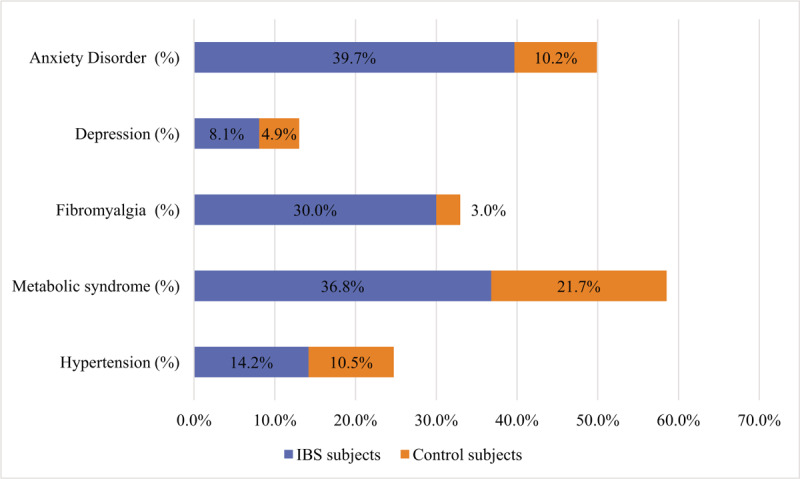
Stacked bar-chart depicting prevalence of anxiety disorder, depression, fibromyalgia and metabolic syndrome in irritable bowel syndrome and control groups.

**Figure 3:**
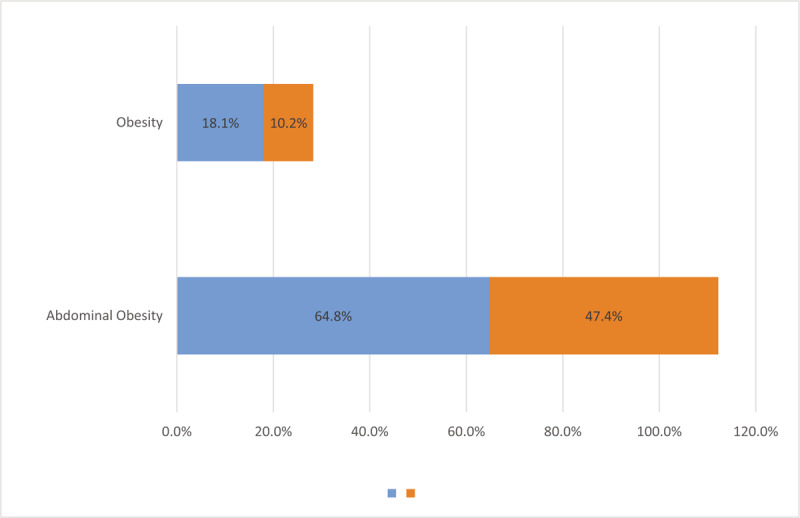
Stacked bar-chart depicting prevalence of obesity and abdominal obesity in irritable bowel syndrome and control groups.

IBS patients had significantly higher prevalence of metabolic syndrome compared with control subjects (36.8% vs 21.7%, respectively, *P* < .001) (Fig. 2). The IBS group had higher BMI values than the control group. The rate of obesity was found to be 18.1% among IBS subjects, whereas this value was 10.2% for the control group. Waist circumference was significantly higher in the IBS patients than the control subjects, as well. Abdominal obesity rates were 64.8% and 47.4% in IBS patients and control subjects, respectively (Table 4 and Fig. 3). IBS subjects also had higher degrees of insulin resistance compared to the control subjects, which was evaluated via HOMA-IR levels (1.9 [1.4–3.6] vs 1.7 [1.2–2.9], respectively, *P*:.001). The prevalence of the diagnoses of type 2 diabetes mellitus and hypertension were not different between the groups, however, systolic and diastolic blood pressures as well as fasting plasma glucose and HbA1c levels were significantly higher in the IBS group than the control group. Although more patients were already on statin treatment in the IBS group, triglycerides and low-density lipoprotein cholesterol levels were higher, and HDL cholesterol level was lower in IBS subjects compared with those of control subjects. The differences were statistically significant (Table 2). It was investigated whether the presence of FMS, anxiety disorder, or depression affect the development of metabolic syndrome independent of IBS. Both single and multiple logistic regression models indicated that the presence of FMS, anxiety disorder, and depression had no effect on the development of metabolic syndrome (*P* > .05 for each) (Table 5).

**Table 4:**
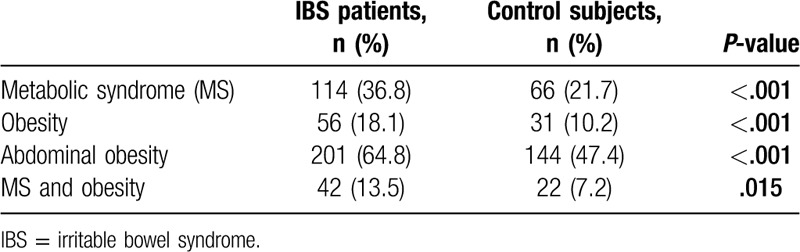
Metabolic syndrome, obesity, and abdominal obesity distribution in the IBS and the control groups.

**Table 5:**
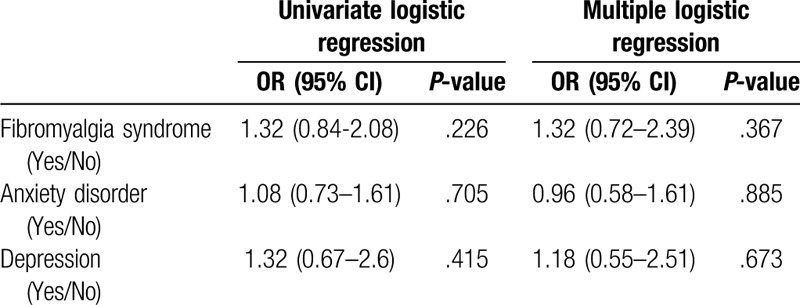
Univariate and multiple logistic regression analyses of possible risk factors for the development of metabolic syndrome.

### 3.3 Fibromyalgia Syndrome, Anxiety Disorder, and Depression

The rate of fibromyalgia was significantly higher in the IBS group than the control group (30% vs. 3%, respectively, *P* < 0.001). Anxiety disorder was detected 39.7% of the IBS subjects compared with 10.2% of the control group (*P* < 0.001). Depression was diagnosed via HDRS in 8.1% of the IBS subjects, whereas this rate was 4.9% among control subjects (*P* < .001) (Fig. 2).

## 4 Discussion

The main finding of the present study was that metabolic syndrome prevalence was significantly higher in patients with IBS compared with the control subjects. To the best of our knowledge, this is the first study in the literature looking at the frequencies of depression/anxiety and FMS while exploring the relationship between IBS and MetS. Both conditions were significantly more common in the IBS patient group compared with the controls. Besides, both general and abdominal obesity was more prevalent among IBS patients compared with the control subjects.

To date, there has been a paucity of data in the literature regarding the relationship between IBS and metabolic syndrome. In a population-based study, Guo et al found that the odds ratio of having metabolic syndrome among patients with IBS was 2.01 (CI, 1.13–3.55). The authors reported a significantly higher prevalence of metabolic syndrome in IBS patients compared with non-IBS subjects.^[10]^ In another retrospective, case-control study, Lee et al demonstrated that the prevalence of metabolic syndrome was 52.3% in obese IBS patients and 10.3% in nonobese IBS patients. Both rates were significantly higher compared with the control subjects.^[11]^ We found the prevalence of metabolic syndrome among the patients with IBS as 36.8% compared to 21.7% in the control subjects (*P* < .001). Our results also showed a significant difference in the rates of metabolic syndrome between obese and non-obese metabolic syndrome patients.

In their comprehensive review, Pickett-Blakely and colleagues reported the prevalence of IBS among obese adults as between 11.6% and 24%.^[15]^ Some, but not all, studies evaluated in this review suggested a relationship between obesity and IBS frequency. Obesity leads to a change in the composition of gut microbiota; this alteration, in turn, could increase the number of calories extracted from ingested food and produce functional symptoms that are defined as IBS.^[16]^ Visceral adipose tissue was suggested as another important contributory factor for the development of IBS.^[17,18]^ Visceral adipose tissue is an important source of cytokines, which can lead to increased systemic inflammation as well as gut inflammation. Other potential pathophysiologic mechanisms put forward to account for the relationship between obesity and IBS are nutrition, and psychological factors.^[19,20]^ In our study, IBS patients had significantly higher BMI and waist circumference levels compared with the control subjects. Thus, in our opinion, one of the primary reasons for more frequent MetS among IBS patients was the fact that IBS patients were generally and abdominally more obese. In addition, MetS prevalence among obese and nonobese IBS patients was 75% and 28%, respectively. Our results were in line with the aforementioned 2 studies. The frequency of metabolic syndrome in nonobese IBS patients was close to that of the control subjects. These findings, once again point to the crucial role of obesity for metabolic syndrome risk. Interestingly, although it seems counterintuitive, there were no differences in terms of general and abdominal obesity across the IBS subgroups.

Obesity causes metabolic syndrome not only through insulin resistance but also via alterations in gut microbiota. Gut microbiota in obese individuals demonstrates notable changes from the microbiota observed in lean individuals.^[21]^ This altered microbiota leads to delayed satiety, increased inflammatory cytokines, increased lipogenesis in the liver, decreased GLP-1 production, and decreased butyrate production.^[22]^ Consequently, obese individuals become prone to the development of the metabolic syndrome. Several studies have consistently demonstrated that patients with IBS have altered gut microbiota.^[23,24]^ Thus, pathophysiologically, it is plausible to consider that in obese individuals, altered gut microbiota might lead to the development of both irritable bowels syndrome and metabolic syndrome. Recent studies, including ours reporting the high frequency of metabolic syndrome in patients with IBS lend support to this association.^[10,11]^

In a recent meta-analysis, selfreported anxiety and depression were found to confer a 2-fold risk increase for the development of IBS.^[25]^ Included in this systematic review were prospective cohort and case-control studies; hence, the presence of depression and or anxiety emerged as significant factors for IBS onset. Thus, increased depression and anxiety, through IBS, may have some impact on the development of the metabolic syndrome. Since both are described as functional disorders, it would not be surprising to note that IBS frequency is increased among fibromyalgia patients and vice-versa. Yang et al found that the presence of fibromyalgia increased the risk of IBS 1.54-fold.^[26,27]^ To the best of our knowledge, ours is the first study to evaluate the prevalence of depression, anxiety disorder, and FMS in association with metabolic syndrome in IBS patients. Our results also confirmed the previous findings in the literature in this regard. Depressive symptoms, anxiety disorder, and FMS were significantly more common among IBS patients than the control subjects. Especially, depression and anxiety should be meticulously evaluated in patients with IBS, because these disorders have the potential to initiate IBS symptoms, and through which might lead to the development of metabolic syndrome components.

Some limitations of the present study deserve mention. Firstly, depression and anxiety disorder diagnoses were made based on some inventories rather than clinical evaluation by a physician. Secondly, we did not take into account the dietary differences likely to occur between the groups. On the other hand, our study has several strengths; firstly, the screened population was relatively large and there were sufficient subjects in each group to allow comparisons. Secondly, we vigorously excluded potential underlying disorders in the IBS group.

In conclusion, this present study has shown a significantly increased rate of metabolic syndrome among IBS patients than the control subjects. Both abdominal and general obesity was significantly more common in IBS patients, as well. Depression, anxiety, and FMS were also more frequent among IBS patients compared with the controls. Further studies are clearly needed in this currently evolving research topic.

## Acknowledgment

The author would like to thank biostatistician Mr. Gökhan Karakoç for statistical consultation.

## Author contributions

**Conceptualization:** Muharrem Bayrak.

**Data curation:** Muharrem Bayrak.

**Formal analysis:** Muharrem Bayrak.

**Investigation:** Muharrem Bayrak.

**Methodology:** Muharrem Bayrak.

**Visualization:** Muharrem Bayrak.

**Writing – original draft:** Muharrem Bayrak.

**Writing – review & editing:** Muharrem Bayrak.
